# Effort and salience jointly drive saccade selection

**DOI:** 10.3758/s13423-025-02701-w

**Published:** 2025-05-15

**Authors:** Damian Koevoet, Christoph Strauch, Marnix Naber, Stefan Van der Stigchel

**Affiliations:** Experimental Psychology, Helmholtz Institute, Utrecht University, Utrecht, The Netherlands

**Keywords:** Effort, Cost, Salience, Saccade selection, Attention

## Abstract

Choosing where to move the eyes (’saccade selection’) is one of the most frequent human decisions and fundamentally shapes perception. Currently, saccade selection is thought to be predominantly driven by the observer’s goals, selection history, and by the physical salience of stimuli. Recent work demonstrates that the inherent effort associated with planning and executing saccades (’saccade costs’) also drives saccade selection: participants prefer making affordable over costly saccades. Do saccade costs still affect saccade selection when other factors such as salience attract gaze? Here, we addressed if, and how, saccade costs and salience together drive saccade selection by having participants freely choose between two potential saccade targets in different directions. Saccade targets either differed in salience or not, allowing us to disentangle the effects of saccade costs and salience. We observed that salience predicted saccade selection: participants chose salient over non-salient targets. Furthermore, saccade costs predicted saccade selection when equally salient targets were presented. When the possible targets differed in salience, the effect of saccade costs on saccade selection was reduced but not eliminated. Further analyses demonstrate that saccade costs and salience jointly drive saccade selection. Together, our results are in line with an accumulating body of work, and show that the role of effort in saccade selection is robust to salience. We conclude that effort must be considered a fundamental factor that drives where the eyes are moved.

**Supplementary Information:**

The online version contains supplementary material available at 10.3758/s13423-025-02701-w.

## Introduction

Humans and other foveal animals make ballistic, jerk-like eye movements, called saccades, to inspect objects in the environment at a high acuity (Findlay & Gilchrist, 2003). As humans execute *∼*3–4 saccades per second (Henderson, 2003; Henderson & Hollingworth, 1998), the selection of the upcoming saccade target is one of the most frequent decisions the brain is faced with (Bargary et al., 2017; Tatler et al., 2017).

What determines which saccade target is selected? The tripartite model of attention posits that visual selection is driven by the observer’s goals, selection history, and the physical salience of stimuli (for reviews, see Awh et al., 2012; Theeuwes et al., 2022). Although this powerful model explains many existing findings, it does not capture systematic biases in oculomotor behavior such as a persistent central bias or a pronounced preference for cardinal over oblique saccade directions (Anderson et al., 2008; Burlingham et al., 2024; Engbert & Kliegl, 2003; Foulsham & Kingstone, 2010; Foulsham et al., 2008; Tatler & Vincent, 2009). What can account for these systematic eye-movement biases? These and other unexplained oculomotor behaviors may be explained by saccade costs, defined as the intrinsic effort of planning and executing saccades (Hoppe & Rothkopf, 2016, 2019; Kadner et al., 2022; Koevoet et al., 2025b; Koevoet et al., 2023; Thomas et al., 2022).

Although an extensive literature uses the term “saccade cost,” the term has been used to mean many different things. For example, the term has been used to mean having less time available to sample information or a sense of “urgency” (Churchland et al., 2008; Sedaghat-Nejad & Shadmehr, 2021), the (energy) efficiency of the resulting movement itself (Harris & Wolpert, 1998; Lisi et al., 2019), extrinsic consequences (e.g., punishment, reward, or memory load) associated with specific eye movements or targets (Moskowitz et al., 2023; Platt & Glimcher, 1999; Schut et al., 2017; Shadmehr et al., 2010; Wagner et al., 2023; also see Schall, 2001), or the effort associated with the decision of where to sample information itself (Araujo et al., 2001; Petitet et al., 2021). Although all of these factors can be interpreted as a cost associated with saccades, here we use the term “saccade costs” to mean the intrinsic effort of planning and executing the saccade itself. In this context, saccade costs are explicitly independent of *extrinsic* saccade costs, including a saccade’s perceptual consequences, the decision of where to saccade, and the outcomes (i.e., reward, punishment) associated with the eye movement itself. Such intrinsic saccade costs have earlier been speculated to be a driver of the decision to sample externally (with an eye movement) or internally (from working memory; Ballard et al., 1995; Hoogerbrugge et al., 2023; Kibbe & Kowler, 2011; Somai et al., 2020). Our conceptualization of saccade costs are also in line with previous work using computational modeling and/or inferences from gaze behavior itself to quantify saccade costs (Diamond et al., 2017; Hoppe & Rothkopf, 2016, 2019; Kadner et al., 2022; Thomas et al., 2022). Thus, based on an extensive body of work (Ballard et al., 1995; Diamond et al., 2017; Hoogerbrugge et al., 2023; Hoppe & Rothkopf, 2016, 2019; Kadner et al., 2022; Kibbe & Kowler, 2011; Koevoet et al., 2025b; Koevoet et al., 2023; Somai et al., 2020; Thomas et al., 2022; also see Shadmehr & Ahmed, 2020), here we use the term “saccade costs” to mean the intrinsic effort of planning and executing saccades.

While intrinsic saccade costs are likely subtle (Findlay & Gilchrist, 2003; Helmholtz, 1866; Theeuwes, 2012), they can be quantified physiologically using pupil size (Koevoet et al., 2023). Pupil size is a well-established physiological marker of mental (and physical) effort when controlling for low-level visual features (Beatty, 1982; Kahneman, 1973; Koevoet et al., 2024; Mathôt, 2018; Richer & Beatty, 1985; Sirois & Brisson, 2014; Strauch et al., 2022; van der Wel & van Steenbergen, 2018). Whenever one exerts relatively more effort on a given task, the pupil dilates (Beatty, 1982; Bumke, 1911; Kahneman, 1973; Loewenfeld, 1958), allowing for the measurement of saccade costs (Koevoet et al., 2023). As such, we showed that the pupil dilates more during saccade planning compared with covert shifts of attention, demonstrating that saccades are more costly than covert shifts. This is likely due to spatial remapping, presaccadic shifting, and motor preparation of the ensuing movement (Koevoet et al., 2023). The costs of different types of saccades also differ: Oblique saccades have a higher cost than cardinal saccades, and downward saccades are more costly than upward saccades (Koevoet et al., 2025b). In contrast to extant work wherein saccade costs were inferred using computational modelling or gaze behavior itself (Hoppe & Rothkopf, 2016, 2019; Kadner et al., 2022; Thomas et al., 2022), pupil size allows for a physiological quantification of saccade costs independent of saccade selection itself. Do differences in saccade costs contribute to saccade selection?

The law of least effort states that whenever given a choice, humans and other animals will choose the less effortful option (Hull, 1943; Tsai, 1932) at comparable levels of punishment/reward. This is likely because the brain operates on limited resources (Castrillon et al., 2023; Jamadar et al., 2024), and expenditure of such resources should be minimized whenever possible (Friston, 2010; Just et al., 2003; Shadmehr & Ahmed, 2020). In terms of eye movements, one should therefore choose affordable over costly saccade targets when other factors are controlled for (Kadner et al., 2022; Koevoet et al., 2023; Shadmehr & Ahmed, 2020; Thomas et al., 2022). We recently observed that saccade costs differed across directions, and that such costs indeed robustly predicted which directions participants preferred to saccade to (Koevoet et al., 2025b). Put differently: Participants prefer to saccade to affordable targets.

Until now, the effect of saccade costs on saccade selection has mostly been studied in isolation of the observer’s goals, selection history, and salience (but see, e.g., Kadner et al., 2022; Koevoet et al., 2025b). Therefore it is unknown how saccade costs affect saccade selection when other factors such as the observer’s goals, selection history, and salience exert their influence on saccadic selection. Specifically, salience is widely regarded as an important factor that, perhaps automatically, drives attentional selection (Itti et al., 1998; Theeuwes, 1994; Theeuwes et al., 1998; but also see de Vries et al., 2011; Einhäuser et al., 2008; Gaspelin et al., 2015). For example, salient distractors impair visual search performance (Theeuwes, 1994; Theeuwes et al., 2022) and stimulus properties predict which locations are fixated during free viewing (Itti & Koch, 2001; Kümmerer et al., 2022).

Here, we addressed whether saccade costs would still influence saccade selection in the presence of salient information. Using a saccade choice task, we were able to independently manipulate saccade costs (through directions) and salience (through color hue and luminance). This allowed us to test whether salience and saccade cost information are integrated, or whether one factor eliminates the effect of the other during saccade selection. Furthermore, in case both saccade costs and salience could affect saccade selection, we were interested in *how* the confluence of these factors drive where the eyes are moved.

To foreshadow our findings, saccade costs and salience both drove saccade selection. We observed that salience reduced but crucially did not eliminate the effect of saccade costs on saccade selection. This demonstrates that saccade costs and salience are integrated during saccade selection.

## Methods

### Open practices

The data and analyses scripts are openly available (https://osf.io/9482z/). The study was not preregistered.

### Participants

Twenty participants with normal or corrected-to-normal vision (*M*_age_ = 21.2 years, range: 19–25; 11 women, nine men) took part in the experiment. A power analysis with G*Power (Version 3.1; Faul et al., 2007) was conducted using a previously reported effect size of Cohen’s *d* = 0.729 from Koevoet et al. (2025b), wherein the effect of saccade costs on saccade selection was investigated. Seventeen participants were necessary to reach 0.80 power to detect a significant effect (*α* = 0.05) with a two-tailed, one-sample *t* test. We slightly overshot this number to reach the same sample size as in Koevoet et al. (2025b). All participants were compensated with €8 per hour or course credits. The experimental procedure was approved by the ethical review board of Utrecht University’s Faculty of Social Sciences (24–0382).

### Apparatus and eye tracker

Stimuli were presented using PsychoPy (Version 2021.2.3; Peirce et al., 2019) on an ASUS ROG PG278Q monitor (2,560 × 1,440 px; 100 Hz). The right eye was tracked at 1000 Hz with an EyeLink 1000 + (SR Research, Mississauga, Ontario, Canada).^1^ Participants were positioned 67.5 cm away from the monitor in a chin and headrest. A 9-point calibration and validation was conducted at the start of the experiment and between blocks whenever necessary.

### Procedure

**Fig. 1:**
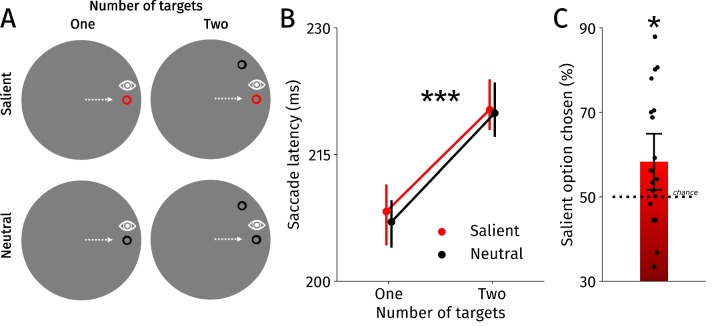
**A** Twenty participants moved their eyes toward a single saccade target, or freely chose between one of two possible saccade targets. Possible targets were either neutral (black) or salient (red). **B** Saccade onset latencies for the saliency and number of target conditions. Error bars indicate bootstrapped within-subject 95% confidence intervals. **C** The percentage of trials wherein the salient over the neutral target was chosen. Black dots represent participants. Error bar holds the bootstrapped 95% confidence interval. *p < 0.05, ***p < 0.001

Participants performed a saccade task wherein a single saccade target was fixated on each trial (based on Koevoet 2025b; Thomas et al., 2022). Throughout the saccade task (Fig. 1A), stimuli were presented on a gray circle (27° diameter, HSV: [0, 0%, 50.2%], *∼*42.5 cd/m^2^) with a black background (HSV: [0, 0%, 0%], *∼*0.18 cd/m^2^) to minimize direction biases as much as possible. Trials started whenever participants fixated a central dot (0.8° diameter) for 100–500 ms (within 3°). Based on the condition, either one or two potential saccade target rings (1° diameter) appeared thereafter at an eccentricity of 10°. There were 36 possible saccade target locations (and therefore directions), which were randomly chosen on each trial with the only criterion that any two locations should be at least 20 radial ° apart from another. Participants were instructed to move their eyes freely to one of the saccade targets. Crucially, one of the saccade targets was presented in red (HSV: [0, 100%, 100%], *∼*42.8 cd/m^2^) in salient trials. All other potential saccade targets were black (HSV: [0, 0%, 0%], *∼*0.18 cd/m^2^). The red saccade targets were considered salient for two reasons: Red targets had higher color hue and higher luminance than black targets (*∼*42.8 cd/m^2^ vs. *∼*0.18 cd/m^2^; see Itti et al., 1998; Itti & Koch, 2001), and red targets were presented less often than black targets, effectively increasing their salience (33.33% vs. 66.67%; see Folk & Remington, 2015; Geyer et al., 2008; Müller et al., 2009). We chose to manipulate salience through color based on extensive visual search (e.g.,Desimone & Duncan, 1995; Feldmann-Wüstefeld et al., 2021; Olivers et al., 2006; Theeuwes, 1994; Theeuwes et al., 2022; van Moorselaar et al., 2014) and computational modeling work (e.g., Itti & Koch, 2001; Itti et al., 1998). Trials ended upon fixation of a saccade target for 100 ms (within 3°), followed by a 350-ms blank interstimulus interval.

We adopted a mixed-design and each condition (see Fig. 1A; Salient, Neutral × One Target, Two Targets) was presented equally often. The first two participants completed 1,440 trials and took breaks after every 120 trials. For the remaining participants, we chose to slightly reduce the number of trials to ensure the experiment would not exceed *∼*60 min—these participants completed 1,152 trials and took breaks every 48 trials.

## Data analysis

Gaze data were epoched from saccade target onset for each trial. During offline saccade detection (based on Koevoet et al., 2023), gaze data were subjected to a Savitzky–Golay filter (Nyström & Holmqvist, 2010; Savitzky & Golay, 1964). Saccade onsets and offsets were detected using velocity thresholds of 50°/s and 1°/s, respectively. Trials with saccade latencies faster than 100 ms or slower than 550 ms were discarded. For the two target conditions (Fig. 1A), the chosen target was defined as the target where gaze was closest based on the last 50 ms of the epoch. Trials where we could not reliably determine which target was chosen (less than 1.5° difference between each target and gaze) were discarded. We also discarded trials where gaze was more than 1.5° away from the target determined to be the chosen option. A total of 1,106 (94.01%) ± 18.15 (0.02%) (mean, s.e.m.) trials were retained per participant.

As an index of saccade costs, we used the saccade costs map that we previously created using a highly similar task (Koevoet et al., 2025b). In Koevoet et al. (2025b), participants performed a delayed-saccade task in 36 different directions, and we recorded pupil size just prior to saccade initiation. As participants were cued to a specific saccade target, pupil size could not have been affected by factors underlying deciding between multiple alternatives (e.g.de Gee et al., 2014; Einhäuser et al., 2010) in the saccade planning task. Moreover, in Koevoet et al. (2025b), we controlled for other possible confounds such as differences in luminance. Our saccade planning task established considerable differences in pupil size during saccade planning across directions. Specifically, the pupil dilated more prior to the execution of oblique compared with cardinal saccades (also see Koevoet et al., 2023), and we observed a larger pupil size prior to downward compared with upward saccades (Koevoet et al., 2025b). Note that we could not create a new saccade cost map from the current data because participants executed saccades as soon as possible, leaving no time for the temporally sluggish pupil to indicate saccade-inherent cost without induced measurement errors (Hayes & Petrov, 2016; Mathôt & Vilotijević, 2022; Strauch et al., 2022). Moreover, in the current data, pupil size could have been contaminated with decision-making-related variables (between multiple variables) that would contaminate the underlying saccade costs signal (e.g., de Gee et al., 2014; Einhäuser et al., 2010).

**Fig. 2:**
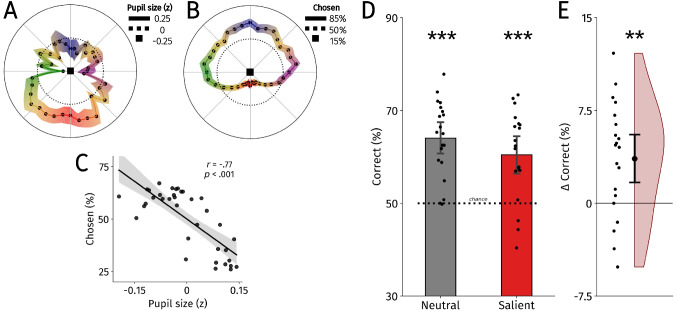
**A** Pupil size in the saccade planning task in all 36 different directions from Koevoet et al. (2025b). **B** The average saccade preferences across all 36 possible saccade target directions collapsed across the neutral and salient conditions. Saccade preferences were calculated by dividing the total amount of times a direction was chosen by the amount of time it was offered as an option. Shaded areas represent the standard error of the mean across participants. **C** Correlation between pupil size during saccade planning (A) and saccade preferences in the current data (B). Individual points represent directions averaged across participants. **D** Saccade cost-based classification performance for the neutral and salient conditions. The *y*-axis reflects the percentage of trials wherein we could classify the chosen target correctly based on saccade cost. Dashed line represents chance level (50%) performance. **E** Differences in saccade cost-based prediction performance between the neutral and salient conditions. Positive values represent better classification performance in the neutral compared with the salient condition. In **A** and **B**, the colors of the error bands represent directions (up = blue; down = red; left = green; right = purple; diagonal = orange). In **D** and **E**, individual points represent participants and error bars hold bootstrapped 95% confidence intervals. **p < 0.01, ***p < 0.001

To assess saccade preferences across directions, we determined multiple direction properties for each potential saccade target. Each target had a specific obliqueness, up-downness and left-rightness score. The cardinal locations (left, right, up, down) had an obliqueness score of 0, and the most oblique locations had a score of 4, independent of the left-rightness or up-downness of the target. The other direction properties follow a similar logic and we used *x* and *y* coordinates for the left-rightness and up-downness scores, respectively, to consider the polar nature of the data. Thus, obliqueness scores ranged from 0 to 4, and the other direction properties ranged from − 10–10°. To analyze preferences between two options, we always subtracted the direction property scores of the nonchosen option from the chosen option. This left us with an average preference for each direction property per participant. If participants had no preference, the resulting subtraction scores should equal 0. Therefore, we analyzed the preference for each direction property using one-sample *t* tests against 0 and we applied a false-discovery rate (FDR) correction using the Benjamini–Hochberg procedure (Benjamini & Hochberg, 1995). To analyze the effect of saccade costs on saccade selection, we performed a simple cost-based classification analysis. For each trial, we determined the saccade costs from both potential saccade targets using the saccade costs map from Koevoet et al. (2025b) (Fig. 2A). Then, for each trial we assumed that participants would choose the saccade target with the smallest associated saccade cost. If participants did not select targets based on cost information, the classification analysis should result in 50% correct. Therefore, we analyzed cost-based classifications using one-sample *t* tests against 50%, and did so separately for the neutral and salient conditions (again FDR-corrected).

All data processing and analyses were performed using custom Python scripts (Version 3.9.1) and JASP (Version 0.18.1). All statistical tests were two-tailed, and we set *α* = 0.05 (see the Supplementary Materials for assumption checks). We specify which statistical test was used prior to each analysis throughout the Results.

## Results

Here, we investigated how saccade cost and salience affected saccade selection. We first used a repeated-measures analysis of variance (ANOVA; 2 Salient, Neutral × 2 One Target, Two Targets) to test whether salience and the number of targets influenced saccade onset latencies. We expected that deciding between two compared with one target would result in longer latencies. Indeed, saccade latencies were significantly longer when deciding between two options, *M*_*two*_ = 220.10 ms, 95% CI [211.86 228.34], *M*_*one*_ = 207.64 ms, 95% CI [201.96 213.32], *F*(1,19) = 32.42, *p* < 0.001, η^2^ = 0.63 (Fig. 1B). Neither the main effect of salience, *M*_*salient*_ = 214.28 ms, 95%-CI [207.75 220.80], *M*_*neutral*_ = 213.46 ms, 95% CI [206.52, 220.41], *F*(1,19) = 1.33, *p* = 0.26, η^2^ = 0.07, nor the interaction effect was significant, *F*(1,19) = 0.57, *p* = 0.46, η^2^ = 0.03. Our analysis indicates that deciding between two targets introduces a more elaborate decision process compared with saccading toward a single target.

As we were primarily interested in how salience and saccade costs predicted saccade selection, all subsequent analyses were conducted on data from the two target conditions exclusively. To test whether salience affected saccade selection, we analyzed whether participants chose the salient option more often than the alternative (one-sample *t* tests against 50%). Therefore, for this specific analysis we only analyzed trials from the salient two-target condition. As expected, participants chose the salient saccade target more often than the non-salient alternative, *M* = 58.30%, 95% CI [51.66%, 64.94%], *t*(19) = 2.39, *p* = 0.027, Cohen’s *d* = 0.53 (Fig. 1C). This established that salience affected saccade selection.

Next, we tested whether saccade preferences differed across directions (Fig. 2A). For each trial, we subtracted the obliqueness, up-downness and left-rightness of the chosen target from the corresponding direction properties of the not-chosen saccade target. We then tested each of these possible asymmetries across directions against zero across participants using one-sample *t* tests collapsed across the neutral and salience conditions. Based on Koevoet et al. (2025b), we expected participants to prefer cardinal over oblique, and upward over downward saccade targets. Indeed, participants preferred cardinal over oblique targets, *t*(19) = 6.18, *p* < 0.001, Cohen’s *d* = 1.38, and preferred upward over downward targets, *t*(19) = 5.89, *p* < 0.001, Cohen’s *d* = 1.32. No significant preference was found between left or rightward targets, *t*(19) = 0.51, *p* = 0.613, Cohen’s *d* = 0.12. Note that these effects were stable in the neutral and salience conditions separately as well (see the Supplementary Materials for details). These analyses showed robust asymmetries in saccade preferences around the visual field.

We then turned to the effect of saccade costs on saccade preferences. As an index of saccade costs, we used the saccade costs map that we created in a previous study using pupil size (see Methods for details; Koevoet, et al., 2025b; Fig. 2A). We created a saccade preference map from the current dataset by averaging preferences across the same 36 directions (Fig. 2B). We tested whether saccade costs predicted saccade preferences by correlating the saccade costs map from Koevoet et al. (2025b; Fig. 2A) and the current saccade preference map (Fig. 2B). If participants preferred affordable over costly saccade directions, the saccade costs and saccade preference maps should correlate negatively. Indeed, saccade costs predicted saccade preferences across directions, *r*(34) = − 0.77, *p* < 0.001 (Fig. 2C). This demonstrates that even though the saccade cost and saccade preference maps were created using different groups of participants, the effect remained robust.

Now that we established independent effects of salience and saccade costs on saccade selection, we addressed how the presence of a salient target affected the weighing of saccade costs during saccade selection. In the analysis, we predicted that participants would choose the more affordable target on every trial (determined using the saccade costs map), and tested the performance of this classification against chance level performance (50%) using one-sample *t* tests. The performance of this classification quantifies how well saccade costs predicted saccade selection (i.e., better classification implies a more pronounced weighing of costs and vice versa). To assess whether the presence of salient saccade target affected the link between saccade costs and saccade selection, we conducted this analysis separately for the salient and neutral conditions. As expected, we found that in the non-salient condition saccade costs predicted saccade selection on a trial-by-trial basis, *M* = 64.04%, 95% CI [60.55%, 67.52%], *t*(19) = 7.70, *p* < 0.001, Cohen’s *d* = 1.72 (Fig. 2C) directly replicating previous work (Koevoet et al., 2025b). We found a similar pattern in the salient condition, *M* = 60.44%, 95% CI [56.42%, 64.46%], *t*(19) = 4.96, *p* < 0.001, Cohen’s *d* = 1.11 (Fig. 2C). We then compared the classification performance between the non-salient and salient conditions, and found that the performance was significantly worse in the salient condition, *t*(19) = 3.49, *p* = 0.002, Cohen’s *d* = 0.78 (Fig. 2D). Thus, the presence of a salient saccade target reduced the effect of saccade costs on saccade preferences, but it did not eliminate it.

**Fig. 3:**
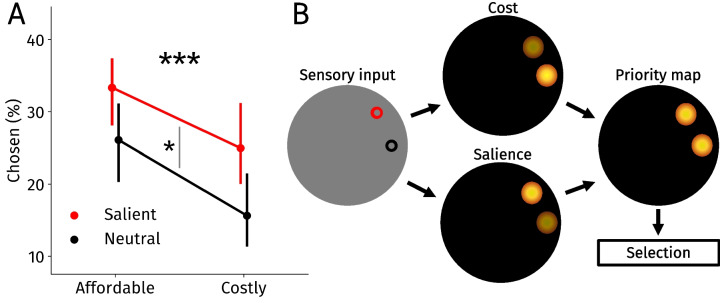
**A** The average proportion of saccade targets chosen split on their saccade cost and salience. Note that the sum of these percentages is 100% for each participant. Error bars indicate bootstrapped within-subject 95% confidence intervals. **B** Schematic overview of our results. Saccade costs (or effort) and salience jointly feed into an attentional priority map. Finally, the priority map is used to decide which target is ultimately selected. *p < 0.05, ***p < 0.001

The above analysis compared conditions wherein participants choose between two non-salient targets with a situation where one target is salient and the other is not. While this comparison shows that salience reduces the effect of saccade selection, it remains unclear how salience and saccade costs jointly drive saccade selection in situations wherein both exert their influence. To ascertain whether saccade costs and salience interact or affect saccade selection independently, we zoomed into trials from the two-target salient condition (Fig. 3A). We first split trials based on whether an affordable or costly target was chosen using a median split on the saccade costs variable. We then further split the data based on whether a salient or neutral target was ultimately chosen. From this, we calculated the percentage of trials in which a certain combination between salient and costliness was ultimately chosen (e.g., salient and costly or neutral and affordable). This allowed us to disentangle the effects of saccade costs and salience on saccade selection when both exerted their influence on each trial.

As expected based on the previous analyses, participants chose salient targets more often than non-salient options, main effect of salience: *F*(1,19) = 5.71, *p* = 0.027, η^2^ = 0.23, and affordable targets were strongly preferred over costly targets, main effect of saccade costs: *F*(1,19) = 28.65, *p* < 0.001, η^2^ = 0.60. Moreover, the interaction effect between saccade costs and salience reached significance, *F*(1,19) = 4.56, *p* = 0.046, η^2^ = 0.19.

To ascertain the robustness of these results, we also ran a generalized linear mixed-effects model (see the Supplementary Material for details). This analysis has the advantage of analyzing saccade selection on a trial-by-trial level without necessitating a median split on the saccade costs variable. Complementing the median-split results, this analysis revealed that the main effects of salience, *β* = 0.82, 95%vCI [0.18,1.47], *t* = 2.50, *p* = 0.012, and saccade costs, *β* = − 5.36, 95% CI [− 7.22, − 3.50], *t* = 5.65, *p* < 0.001, are robust. In contrast to the median-split analysis, the interaction effect between salience and saccade costs did not reach significance, *β* = 0.73, 95% CI [− 0.22, 1.67], *t* = 1.51, *p* = 0.132. Thus, the interaction effect does not seem reliable. Together, our analyses still converge on the conclusion that salience and saccade costs jointly drive saccade selection.

## Discussion

Given that eye movements fundamentally shape perception, it is vital to understand how humans choose the upcoming saccade target. Here, we investigated how saccade costs and salience jointly drive saccade selection. The results showed that participants chose salient saccade targets more often than non-salient options. Furthermore, we found that saccade costs predicted saccade selection. Crucially, saccade costs drove saccade selection even when a salient saccade target was offered. When salience exerted its effect on saccade selection, it reduced but did not eliminate the effect of saccade costs when deciding where to move the eyes. When examining decisions more closely, we found that both salience and saccade costs drove saccade selection (Fig. 3). Our results show that saccade costs and salience are integrated during saccade selection.

The current findings add to a growing body of work emphasizing the role of intrinsic saccade costs during saccade selection (Burlingham et al., 2024; Diamond et al., 2017; Hoppe & Rothkopf, 2016, 2019; Kadner et al., 2022; Koevoet et al., 2025b ; Shadmehr & Ahmed, 2020; Thomas et al., 2022). In contrast to previous work that inferred saccade costs from gaze behavior or through computational modelling (Hoppe & Rothkopf, 2016, 2019; Kadner et al., 2022; Thomas et al., 2022), here we capitalized on the fact that pupil size can physiologically index saccade costs independently from saccade selection (Koevoet et al., 2025b; Koevoet et al., 2023). With access to a direct physiological measure of saccade costs, we demonstrate that these costs drive saccade selection even when salience affects eye movements. This shows that salience does not eliminate the effect of saccade costs on saccade selection, providing novel evidence that effort must be considered a fundamental driver of saccade selection. We see considerable potential in combining physiologically measured and established costs with the assumed costs in computational models of attentional selection (Hoppe & Rothkopf, 2016, 2019; Kadner et al., 2022; Thomas et al., 2022). Together, these lines of work could establish a new class of biologically informed and powerful models of visual attention.

Besides salience, the tripartite model of attention posits that the observer’s goals and selection history drive visual selection. These factors are thought to create an underlying “priority map,” where visual selection is decided in a winner-takes-all fashion (see Fig. 3B; Awh et al., 2012; Theeuwes et al., 2022). In addition to these factors (Awh et al., 2012; Theeuwes et al., 2022), saccade costs must be considered a fourth factor driving saccade selection. Although the interactions between salience, goals and selection history have been investigated intensively (e.g., de Vries et al., 2011; Donk & van Zoest, 2008; Theeuwes, 2010), studies investigating the interactions between saccade costs and other factors are only starting to emerge (Kadner et al., 2022; Koevoet et al., 2025b). Indeed, previous work demonstrated that saccade costs are considered in saccade selection during more natural viewing during which other factors such as salience exerted their influence (Kadner et al., 2022; Koevoet et al., 2025b). We extend these findings here. Instead of natural viewing, here we employed a well-controlled design, which allowed us to disentangle the influences of saccade costs and salience. By using a more controlled design, we were able to investigate the confluence of saccade costs and salience drive saccade selection in more detail (see Fig. 3). More specifically, in our data, the effects of saccade costs and salience did not reliably interact: Salience reduced the effect of saccade costs on saccade selection but did not eliminate it. However, as both salience and cost are continuous factors, there are likely situations where this does not strictly hold: For instance, if a stimulus is extremely salient, saccade costs may not be as predictive of which saccade target is selected. Nevertheless, our results have consequences for models of visual selection and provides insights into how the brain weighs saccade costs during saccade selection. Future work is necessary to understand how the four factors (i.e., goals, selection history, salience, and effort) together shape the underlying attentional priority map that ultimately drives visual selection.

In the current saccade preference task, participants freely chose between two potential saccade targets in the two-target conditions. The two saccade targets always differed in terms of direction, and we observed robust preferences across the visual field. However, after a forward saccade to the target was made, participants had to fixate the center of screen before the next trial started. Previous work has shown that participants are able to plan sequences of multiple saccades (e.g., De Vries et al., 2014; Hoppe & Rothkopf, 2019), and one may argue that participants planned sequences of forward (toward the target) and backward (toward the center) saccades. If this was the case, one would expect no differences in saccade preferences between two adjacent directions, as such sequences would consist of the same two saccades. For example, completely up and downward saccades would require both one up and one downward saccade in both directions, which should lead to minimal preference differences. Instead we found a preference for up over downward saccades, and participants thus likely did not plan saccade sequences in the current task.

Here, we manipulated salience through color hue and luminance to be consistent with a large body of existing literature (e.g., Desimone & Duncan, 1995; Feldmann-Wüstefeld et al., 2021; Olivers et al., 2006; Theeuwes, 1994; Theeuwes et al., 2022; van Moorselaar et al., 2014). However, other visual features such as contrast, luminance and size (and others) also affect visual salience (Itti & Koch, 2001; Itti et al., 1998). As other visual features guide visual selection in a comparable fashion to color (e.g., de Vries et al., 2011; Duncan & Theeuwes, 2024; Itti & Koch, 2001; van Heusden et al., 2023), different manipulations of salience should result in similar effects, but generalization across other visual features, such as contrast and orientation, remains to be tested in future work.

What determines the cost of a saccade? Although pupil size sensitively tracks saccade costs, in isolation it will be unable to inform about what underlies differences in costs (see Koevoet et al., 2025b, for a detailed discussion). We speculate that the intrinsic cost of planning and executing a saccade is a combination of the saccade’s motor and attentional components, as well as its metrics (i.e., latency, amplitude, etc.; Koevoet et al., 2025b; Koevoet et al., 2023). As for the motor component, differences in the complexity of oculomotor programming may contribute to saccade costs. This may explain why oblique saccades are more costly than cardinal saccades, because integrating horizontal and vertical saccade vectors is more complex neurally (Curthoys et al., 1984; King & Fuchs, 1979; Sparks, 2002). Moreover, it is possible that differences in how presaccadic attention is deployed around the visual field underlie saccade costs. As such, presaccadic attention seems to be shifted differently when preparing up compared with downward saccades, which could underlie this difference (Hanning et al., 2022, 2024; but see Koevoet et al., 2025a). Importantly, the early visual cortex also shows a considerable vertical asymmetry, where the lower visual field is represented more strongly than the upper visual field (Benson et al., 2021; Himmelberg et al., 2021, 2022; Silva et al., 2018; Van Essen et al., 1984; also see Himmelberg et al., 2023), which could also underlie the up–down difference in saccade costs. Besides direction, other properties of a saccade may also contribute to its cost, such as its amplitude, velocity, vigor and/or latency (Hoppe & Rothkopf, 2019; Koevoet et al., 2023; Moresi et al., 2008; Naber & Murphy, 2020; Shadmehr & Ahmed, 2020; Wang et al., 2017, 2021; also see Cos et al., 2011, 2012, 2014), but direct research is necessary to understand the link between saccade metrics and pupil size. Thus, we speculate that saccade costs determined by a confluence of motor and attentional components, underlying perceptual, attentional and cortical asymmetries, as well as properties of the saccade itself. Revealing the origins of saccade costs is instrumental in understanding how saccade costs drive saccade selection.

The current paper focused on saccade selection, but visual selection also occurs covertly without overt saccadic eye movements (Carrasco, 2011; Helmholtz, 1866; Posner, 1980). It remains open whether covert attentional shifts costs show a comparable costs pattern across directions and how these interact with factors such as salience. Moreover, it is unknown whether covert attention incorporates attentional shift costs in the way that saccade selection does. Pupil size is able to measure the costs of covert attentional shifts too (Koevoet et al., 2023), which allows for tackling these open questions. We hypothesize that attentional costs also play a role when choosing where to deploy covert attention, but future work is needed to test this directly.

To conclude, our results show that saccade costs and salience both exert their influence on the priority map and jointly drive saccade selection (Fig. 3B). Our findings are in line with an accumulating body of work that demonstrates the vital role of intrinsic saccade costs in saccade selection (Hoppe & Rothkopf, 2016, 2019; Kadner et al., 2022; Koevoet et al., 2025b; Thomas et al., 2022). Together, we conclude that the tripartite model of saccade selection must be extended to a quadripartite model: Saccade selection is driven by the observer’s goals, selection history, physical salience, and cost.

## Supplementary Information

Below is the link to the electronic supplementary material.

Supplementary file1 (PDF 285 KB)

## Data Availability

Data and analyses scripts to reproduce the results are available via the Open Science Framework: https://osf.io/9482z/

## Footnotes

1 Note that one participant was tracked binocularly at 2000 Hz. To be more comparable with other participants, we downsampled this dataset to 1000 Hz and only data from the right eye were used.

## Code availability

Data and analyses scripts to reproduce the results are available via the Open Science Framework: https://osf.io/9482z/
