# Supplementary material for: Effort and salience jointly drive saccade selection
Source: Psychon Bull Rev. 2025 May 15;32(5):2363–74. doi: 10.3758/s13423-025-02701-w (PMC12425846; doi:10.3758/s13423-025-02701-w)
Supplement: Supplementary file 1 — Supplementary file1 (PDF 285 KB) [file 13423_2025_2701_MOESM1_ESM.pdf]

Supplementary Materials to: *Effort and Salience Jointly Drive Saccade Selection*

Damian Koevoet+*, Christoph Strauch+, Marnix Naber, and Stefan Van der Stigchel

Experimental Psychology, Helmholtz Institute, Utrecht University, The Netherlands

+Shared authorship

*Correspondence to d.koevoet@uu.nl

Assumptions for statistical analyses

For all *t*-tests (one-sample and paired) reported in the main paper, we tested whether distributions were approximately normal using Shapiro-Wilk tests. For all tests this was the case – only the distribution for the one-sample t-test of the oblique/cardinal preference was significantly non-normal ($p = .013$; although note that this violation of normality was not apparent from visual inspection). To ensure our effect was not driven by the violation of the assumption of normality, we also ran a non-parametric alternative, the Wilcoxon signed-rank test, which yielded the same conclusion: participants preferred cardinal over oblique saccade targets, $W = 8.0$, $p < .001$. For the repeated-measures ANOVAs we did not test assumptions. Note that for the saccade latency analysis, within-subjects bootstrapped 95% confidence intervals did not overlap (Figure 1B), showing a clear difference between conditions irrespective of distribution assumptions. As for the last analysis (Figure 3A), we also ran a generalized linear mixed-effects model which captured the nested and continuous data structure more adequately to examine the robustness of the effects, for which we provide assumption checks below.

Saccade preferences in neutral and salient trials

In the main paper, we analyzed asymmetries across direction collapsed across the neutral and salient conditions. Here, we provide these results separately for the neutral and salient conditions (Supplementary Figure 1).

As in the main paper, we determined the obliqueness, up-downness and left-rightness for each saccade target (see Methods for details). We then tested whether participants preferred a specific direction property using one-sample *t*-tests. The analyses showed that participants preferred cardinal over oblique saccade targets in the neutral ($t(19) = 3.92$, $p < .001$, Cohen's $d = .88$) and salient ($t(19) = 5.07$, $p < .001$, Cohen's $d = 1.13$) conditions. Moreover, participants preferred up- over downward saccade targets in each condition (neutral: $t(19) = 6.28$, $p < .001$, Cohen's $d = 1.40$; salient: $t(19) = 5.26$, $p < .001$, Cohen's $d = 1.18$). As in the collapsed map, no preference was apparent for left or rightward saccade targets in neither condition (neutral: $t(19) = 0.36$, $p = .72$, Cohen's $d = .08$; salient: $t(19) = 0.66$, $p = .52$, Cohen's $d = .14$).

Finally, we also analyzed whether the extend of these asymmetries in saccade preferences differed between conditions. To this end, we used paired-sample *t*-tests to analyze differences in asymmetries between conditions. Participants preferred up- over downward targets more in the neutral than the salient condition ($t(19) = 2.97$, $p = .008$, Cohen's $d = .66$). The preferences for cardinal vs. oblique ($t(19) = 0.16$, $p = .87$, Cohen's $d = .04$) and left vs. rightward saccades ($t(19) = 0.61$, $p = .55$, Cohen's $d = .14$) did not differ significantly between conditions. Together, these results indicate that salience primarily reduced the preference for upward targets, but not the preference for cardinal saccade targets.

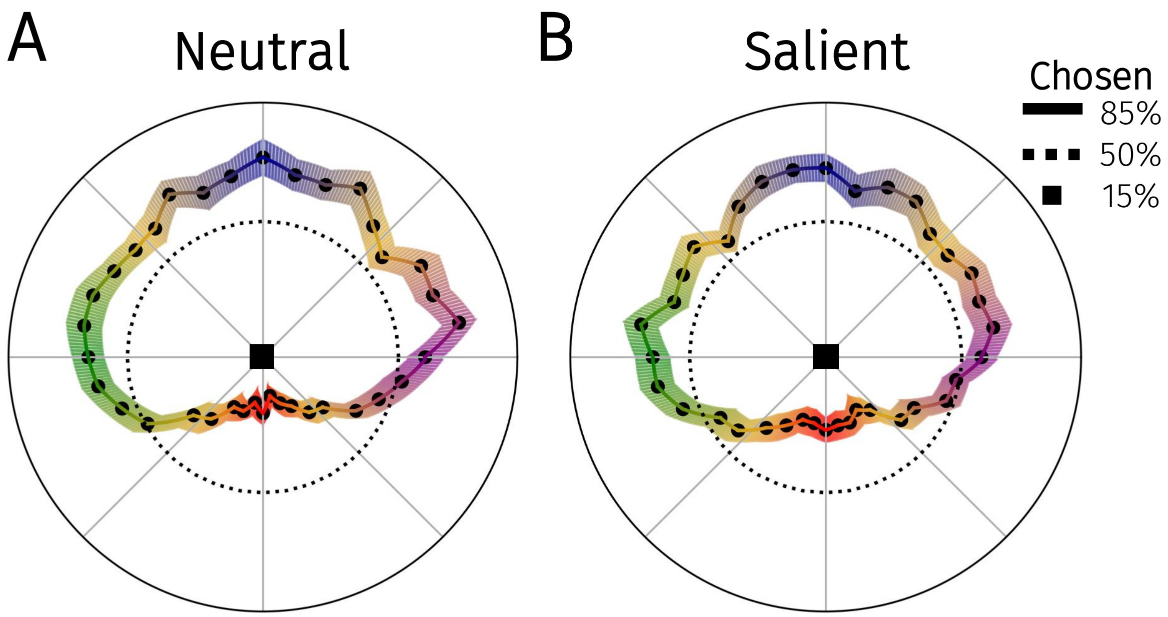

Supplementary Figure 1: Saccade preferences for the **A** neutral and **B** salient conditions. Saccade preferences were calculated by dividing a the amount of times a specific saccade target was chosen divided by how often it was offered.

Generalized linear mixed-effects model

In the main paper, we analyzed how salience and saccade costs affected saccade selection within the two-target salient condition (Figure 4). In that analysis, we used a median-split to create 'cheap' and 'costly' saccade target conditions. This approach loses out on the continuous nature of the saccade costs variable. To account for this, we ran a similar analysis but now using a generalized linear mixed-effects model (similar approach as in Koevoet et al., 2024). Here we provide more details regarding this analysis. We predicted which target was chosen based on its salience and saccade costs, as well as their interaction. We also modeled by-participant intercepts, and random slopes for the main effects of interest (Wilkinson notation: chosen target ∼ saccade costs*salience + (1 + saccade costs+salience)) (Barr, 2013). We also confirmed that the model's assumptions held using the DHARMa package (Hartig, 2024). Specifically, we inspected the QQ plot of the model's residuals which indicated no violations of assumptions (e.g. no outliers/or under/over-dispersion).

References

Barr, D. J. (2013). Random effects structure for testing interactions in linear mixed-effects models. *Frontiers in Psychology*, *4*. https://doi.org/10.3389/fpsyg.2013.00328

Hartig, F. (2024). DHARMa: Residual Diagnostics for Hierarchical (Multi-Level / Mixed) Regression Models.

Koevoet, D., Van Zantwijk, L., Naber, M., Mathôt, S., Van der Stigchel, S., & Strauch, C. (2024). Effort Drives Saccade Selection. *eLife*, *13*. https://doi.org/10.7554/eLife.97760.1
